# Supplementary material for: Synthetic virions reveal fatty acid-coupled adaptive immunogenicity of SARS-CoV-2 spike glycoprotein
Source: Nat Commun. 2022 Feb 14;13:868. doi: 10.1038/s41467-022-28446-x (PMC8844029; doi:10.1038/s41467-022-28446-x)
Supplement: Supplementary file 1 — Supplementary Information [file 41467_2022_28446_MOESM1_ESM.pdf]

Supplementary Information for

Synthetic virions reveal fatty acid-coupled adaptive immunogenicity of SARS-CoV-2 spike glycoprotein

Oskar Staufer, Kapil Gupta, Jochen Estebano Hernandez Buecher, Fabian Kohler, Christian Sigl, Gunjita Singh, Kate Vasileiou, Ana Yagüe Relimpio, Meline Macher, Sebastian Fabritz, Hendrik Dietz, Elisabetta Ada Cavalcanti Adam, Christiane Schaffitzel, Alessia Ruggieri, Ilia Platzman, Imre Berger and Joachim P. Spatz

Correspondence to: imre.berger@bristol.ac.uk, spatz@mr.mpg.de

This document includes:

Supplementary Figures 1 to 15
Supplementary Table 1

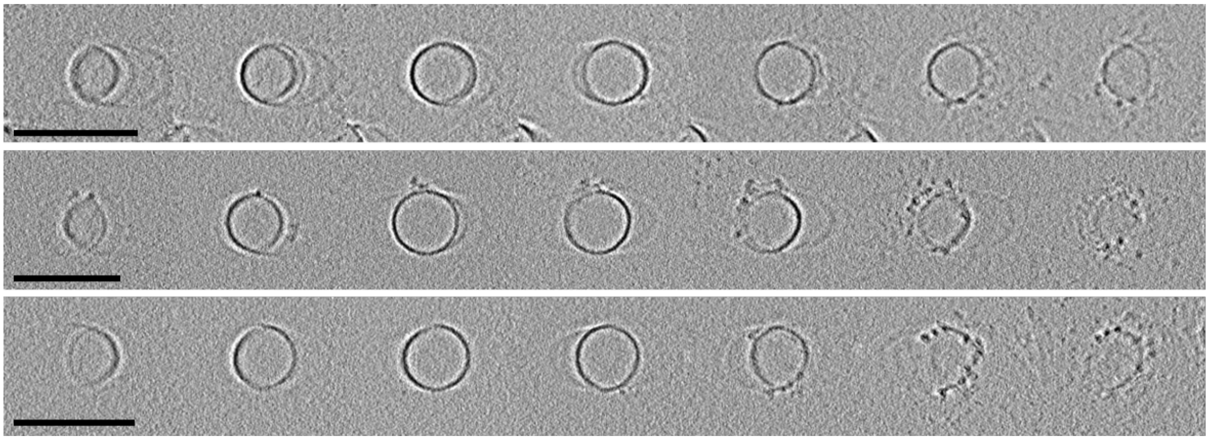

Supplementary Figure 1.

Electron microscopy characterization of MiniVs. Further examples of reconstructed cryoTEM tomography slices of MiniVs with immobilized S on their membrane. Scale bars are 200 nm.

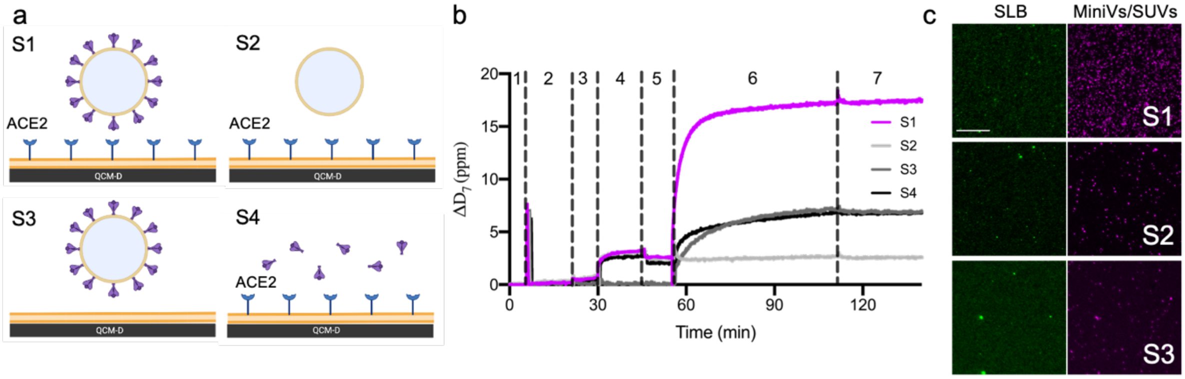

Supplementary Figure 2.

QCM-D Assessment of MiniV ACE2 binding. (**a**) Schematic illustration of experimental conditions (S1-S4) tested for binding of MiniVs with and without S on supported lipid bilayer- (SLB) covered QCM-D quartz crystals presenting recombinant human ACE2 ectodomains. (**b**) Changes in dissipation in 7th harmony ((Δf norm= Δf n/n, with n being the seventh overtone (n=7)) over the whole experimental analysis for S1-S4. Phases correspond to 1. Crystal equilibration in PBS 2. Addition of SUVs and SLB formation with vesicles containing NTA(Ni2+) lipids 3. PBS wash 4. Addition of histidine-tagged recombinant human ACE2 ectodomains or PBS 5. PBS wash 6. Addition of MiniVs, SUVs or soluble S, 7. PBS wash. (**c**) Representative confocal microscopy images, from 2 independent experiments, of the SLBs (green) formed on the QCM-D crystals after experiments from B. MiniVs and SUVs are shown in purple. Scale bar is 10 µm.

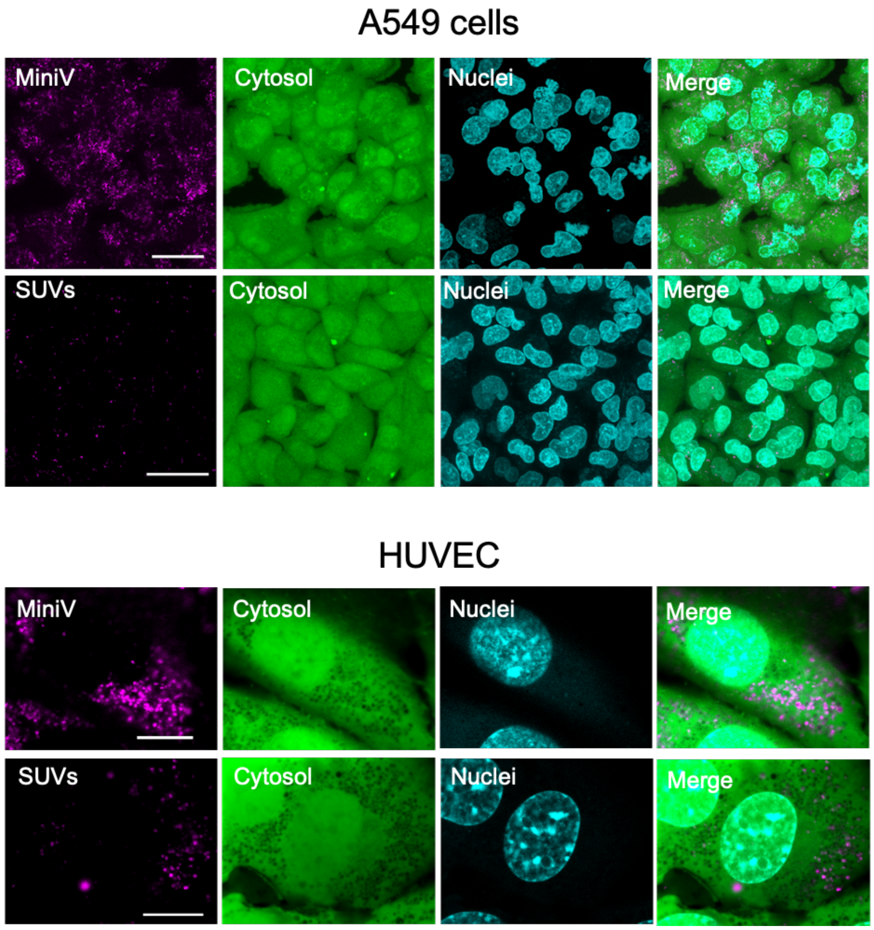

Supplementary Figure 3.
Microscopy analysis of MiniV uptake. Representative confocal microscopy images, from two independent experiments, of MiniV binding and uptake to human alveolar basal epithelial cells (top) and human umbilical vein endothelial cells (bottom) after incubated for 8 hours with Scale bars are 30 µm (top) and 10 µm (bottom).

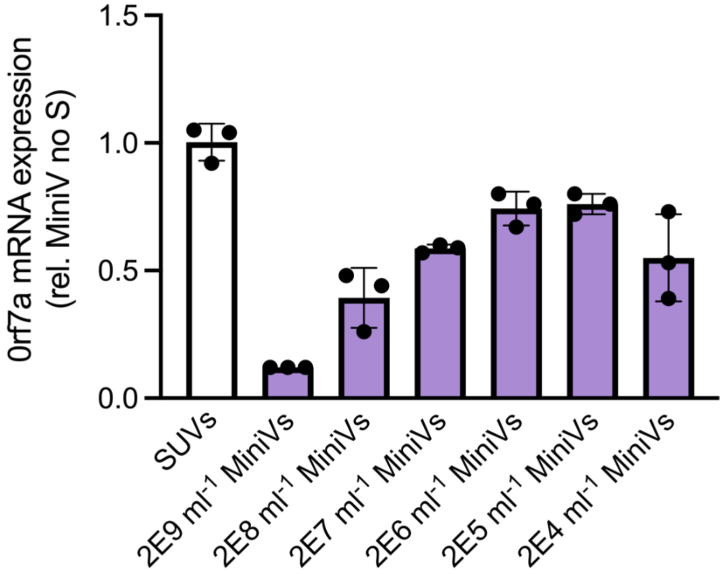

Supplementary Figure 4.

MiniV SARS-CoV-2 competition assays. Serial dilution of SARS-CoV-2 S decorated MiniVs were pre-incubated with ACE2-expressing A549 cells for 2.5 h and subsequently infected with SARS-CoV-2 natural viruses with a multiplicity of infection of 1. Eighteen hours post-infection, ORF7a mRNA expression levels were quantified by qRT-PCR and normalized to infection levels under competitive incubation with SUVs. Results are shown as mean ±SD from n=3 technical replicates.

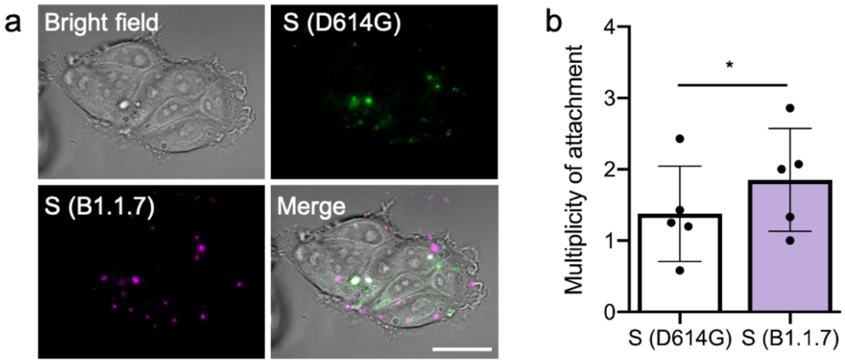

Supplementary Figure 5.

Assessment of emergent variant of concern B1.1.7 MiniVs (**a**) Representative average projection confocal microscopy images of MCF-7 cells incubated for 2 hours simultaneously with MiniVs presenting D614G S (green) or B1.1.7 S (purple). Scale bar is 25 µm. (**b**) Multiplicity of attachment analysis from images in A shown as MiniVs bound per cell. Results from 5 single cell groups are shown. Results are shown as mean ±SD from n=3 biological replicates in each experimental condition, *p<0.05, unpaired two-tailed t-test.

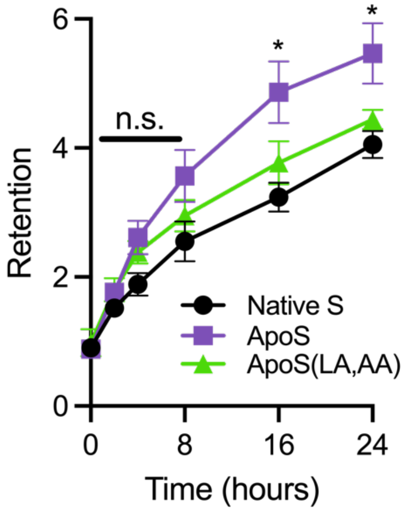

Supplementary Figure 6.
Time resolved retention assays for FFA loading. Time resolved retention assays of MiniVs presenting native S, ApoS or LA and AA-loaded ApoS and incubated with A549 human alveolar basal epithelial cells for 24 hours. Results are shown as mean ±SD from n=3 biological replicates in each experimental condition, *$p<0.05$, n.s. = not significant, unpaired two-tailed t-test.

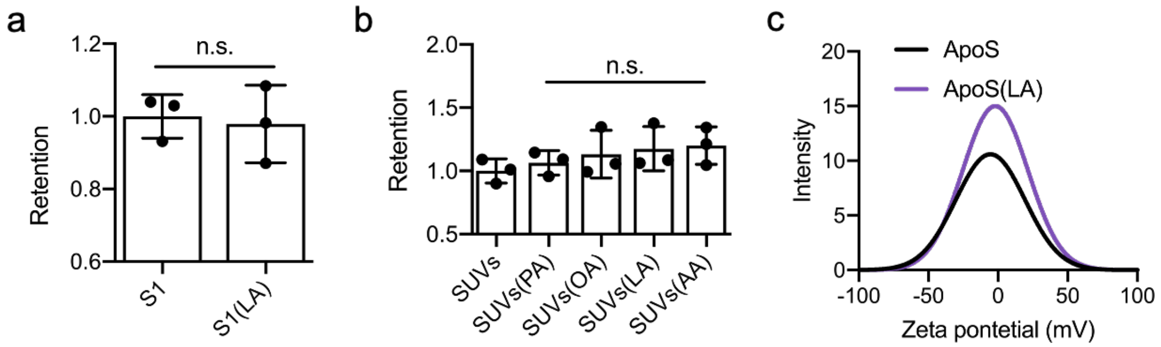

Supplementary Figure 7.
FA loading of SARS-CoV-2 S. (**a**) Retention assay normalized to MiniVs presenting only S1 domains and incubated for 24 h with MCF-7 cells. Addition of LA to S1 domain does not significantly alter MiniV retention. (**b**) SUV-normalized retention assay for SUVs incubated with 25 ng/mL FAs after 24 h incubation with MCF7 cells. (**c**) Zeta-potential distribution analysis by dynamic light scattering of MiniVs presenting ApoS and LA-loaded ApoS in PBS. Results are shown as mean ±SD from n=3 biological replicates in each experimental condition, n.s.= not significant, unpaired two-tailed t-test.

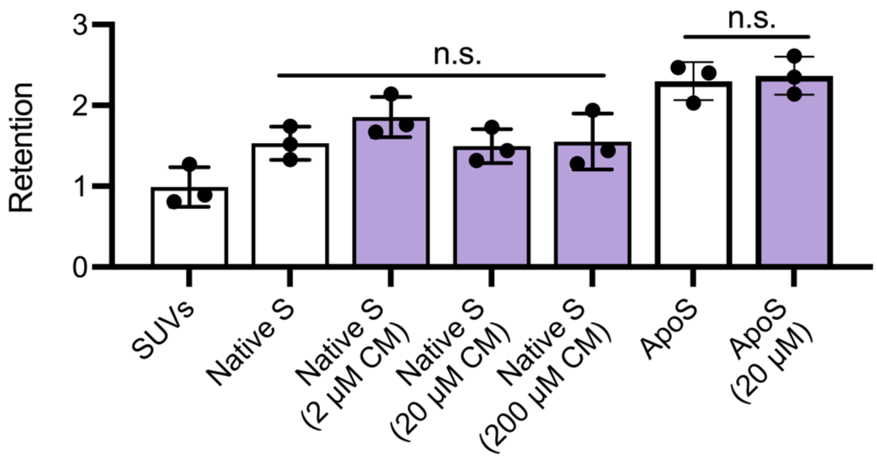

Supplementary Figure 8.
Protease-based S processing. Retention assays for SUVs and MiniVs incubated with A549 human alveolar basal epithelial cells for 8 hours. Retention assays were performed either under untreated control conditions or under treatment with the TMPRSS2 inhibitor camostat mesylate (CM). MiniVs were produced presenting native S or ApoS on the surface. Results are shown as mean ±SD from n=3 biological replicates in each experimental condition, n.s. = not significant, unpaired two-tailed t-test.

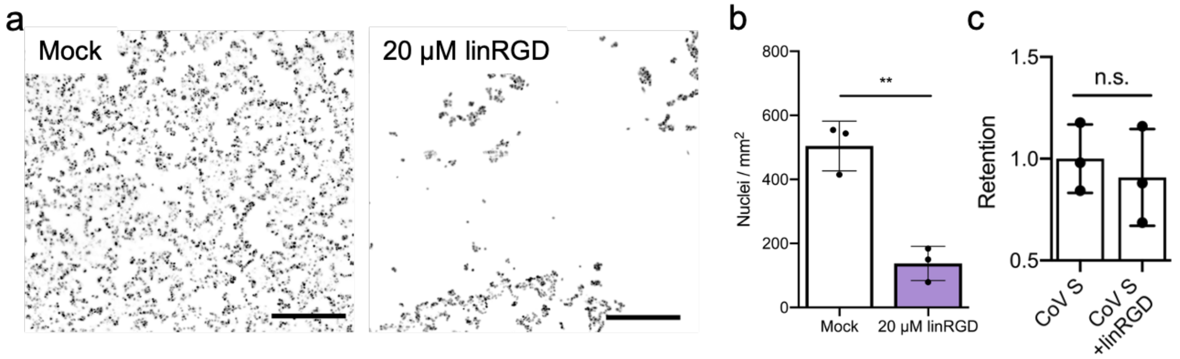

Supplementary Figure 9.
Integrin blocking by linRGD. (**a**) Epifluorescence images of MCF7 cells stained with Hoechst 33342 (inverted fluorescence grey signal) and treated for 24 h with PBS (mock) or 20 µM linRGD. Cell cultures were washed three times before imaging to remove non-adherent cells. Scale bars are 500 µm. (**b**) Cell number quantification by automated counting of nuclei from images in A. (**c**) Retention analysis of MiniVs presenting SARS-CoV S after incubation with MCF7 cells for 24 h. Addition of 20 µM linRGD over the incubation period does not significantly alter MiniV retention. Results are shown as mean ±SD from n=3 biological replicates in each experimental condition, **p<0.005, n.s.=not significant, unpaired two-tailed t-test.

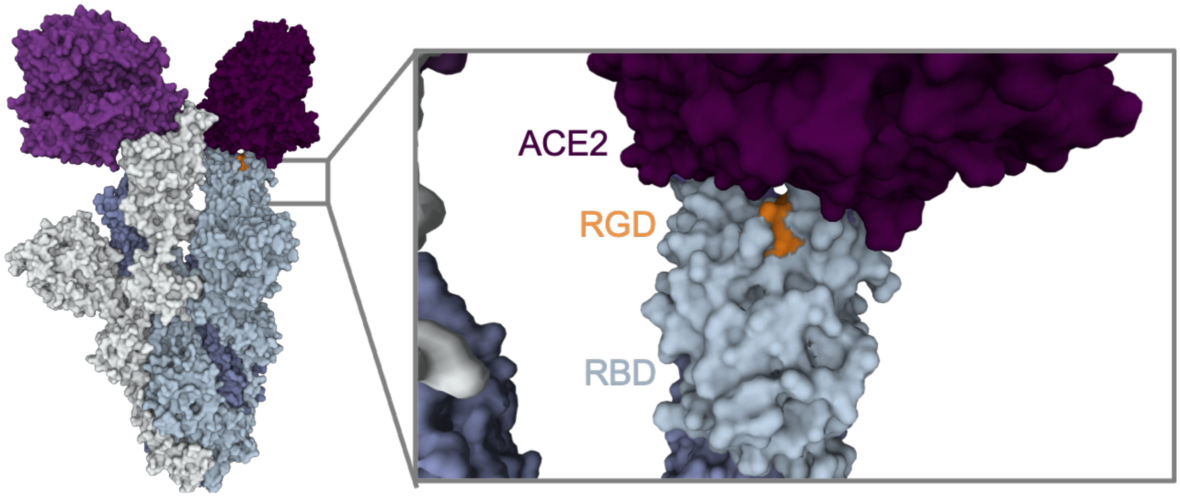

Supplementary Figure 10.
RGD motif presentation in the ACE2-bound S state. Molecular surface representation of the S cryo-EM structure (PDB 7A97) with two open RBDs bound to the ACE2 receptor. ACE2 are shown in purple, RBD in blue and RGD in orange.

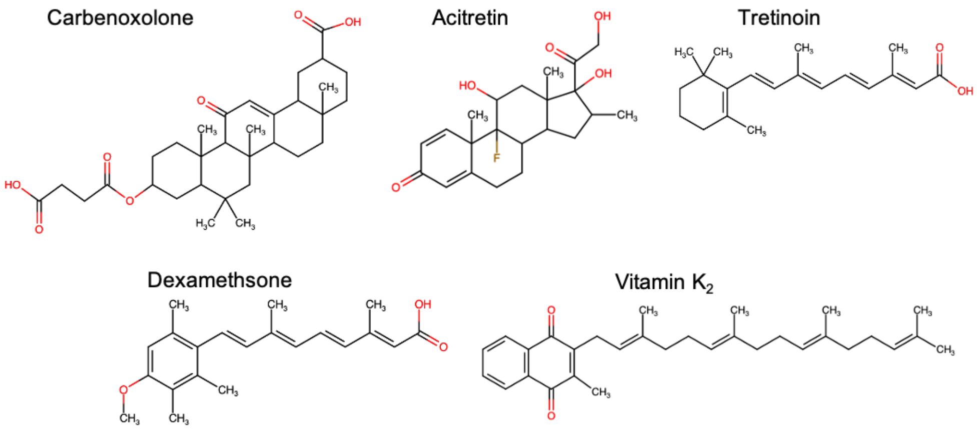

Supplementary Figure 11.
Chemical structures of FDA-approved drugs applied for binding and retention analysis for SARS-CoV-2 S MiniVs.

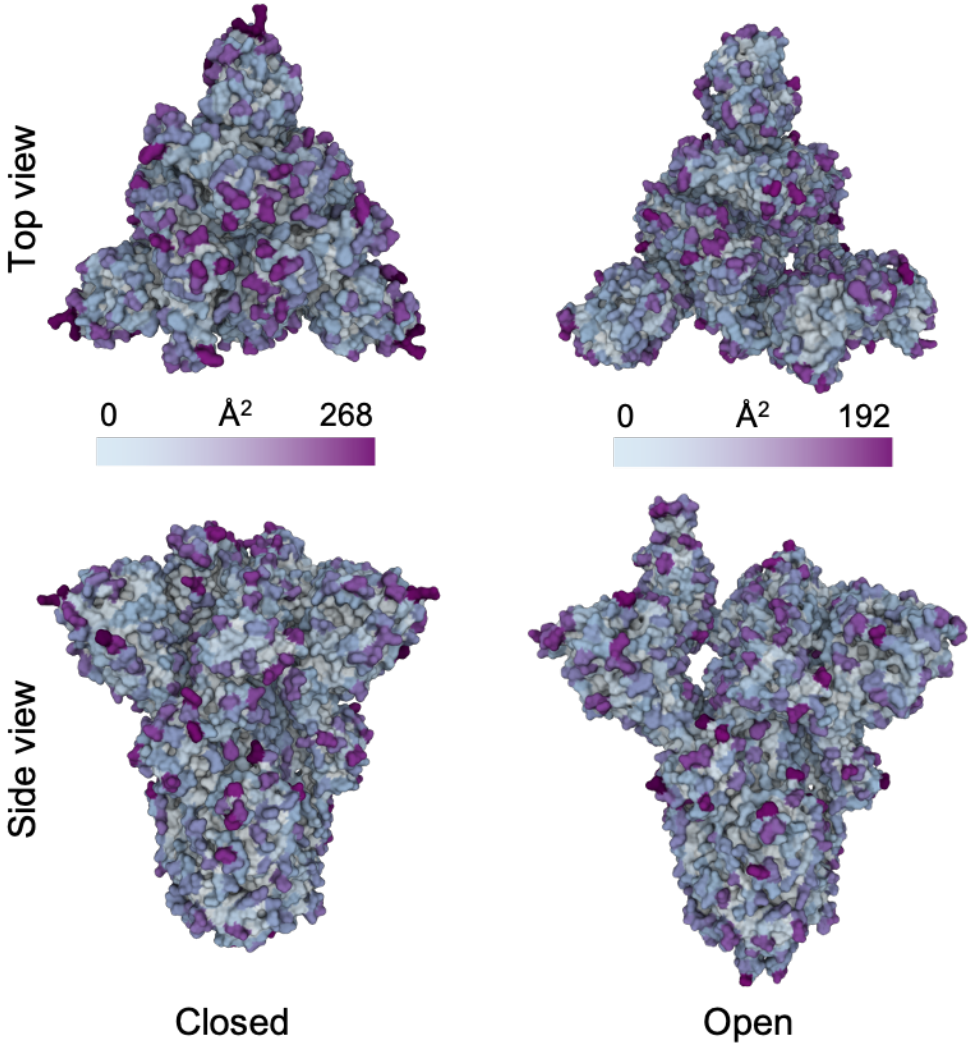

Supplementary Figure 12.
Accessible surface areas of SARS-CoV-2 S. Molecular surface representation of the LA-closed S structure (PDB 6ZB5) (left) and S with one open RBD (PDB 7BNN) (right). Accessible surface areas of individual residues are color-coded.

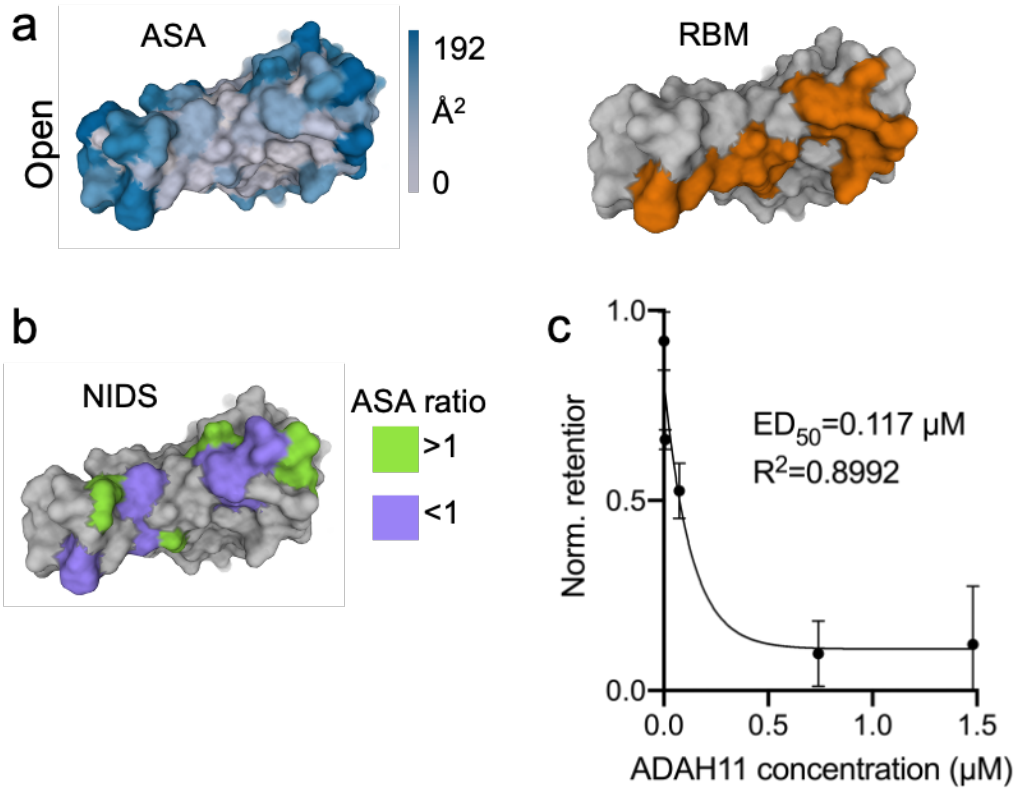

Supplementary Figure 13.
Immunogenicity and neutralization of the RBD. (**a**) Molecular surface representation of the open RBD structure (PDB 7BNN) color coded for accessible surface area of individual residues (left). Molecular surface representation of the open RBD structure (PDB 7BNN) with RBM residues that interact with ACE2 colored in orange (right). (**b**) Molecular surface representation of the open RBD structure (PDB 7BNN). Changes in ASA ratio of NIDS are coloured in green (>1) and purple (<1). (**c**) Minimum-maximum normalized retention assay for MiniVs presenting native S and incubated with MCF-7 cells for 24 hours under treatment with different concentrations of ADAH11 neutralizing nanobody. Curve fitting was performed with a four-parametric sigmoidal fit. Results are shown as mean ±SD from n=3 biological replicates in each experimental condition

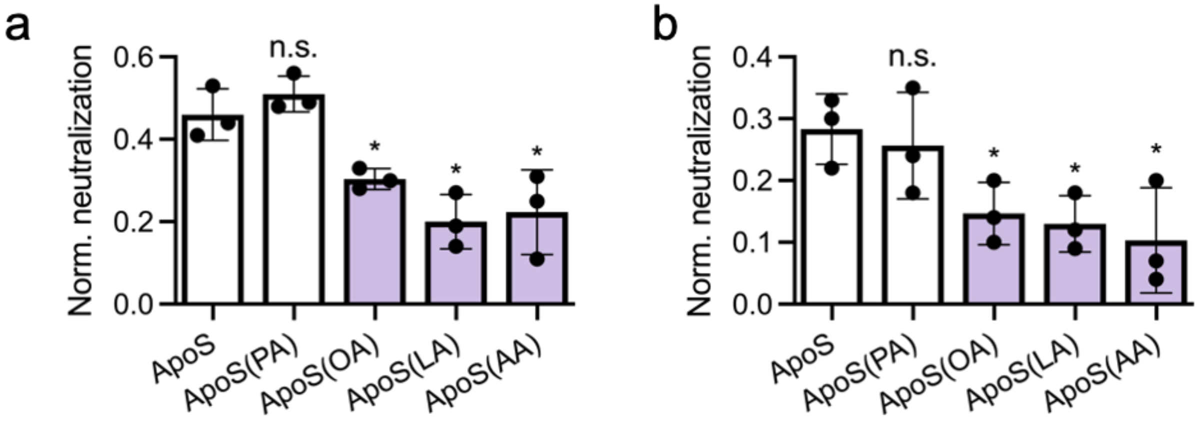

Supplementary Figure 14.
FFA-regulated neutralization potency of IDNS-directed nanobody. (**a**) Native S-normalized neutralization of ADAH11 for MiniVs presenting FFA-loaded ApoS incubated with A549 human alveolar basal epithelial cells for 8 hours. (**b**) Native S-normalized neutralization of ADAH11 for MiniVs presenting FFA-loaded ApoS incubated with human umbilical vein endothelial cells for 8 hours. Results are shown as mean ±SD from n=3 biological replicates in each experimental condition, *p<0.05, n.s. = not significant, unpaired two-tailed t-test.

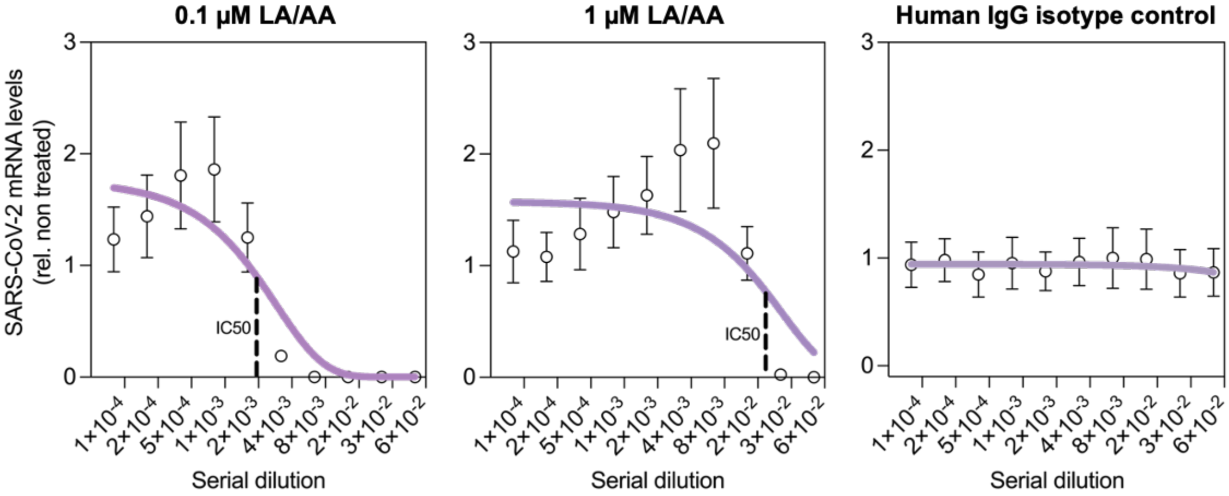

Supplementary Figure 15.

Live virus neutralization assays. Serial dilutions of IgG antibodies were pre-incubated with SARS-CoV-2 natural viruses for 1 h before infection of ACE2-expressing A549 human alveolar basal epithelial cells. Eight hours post-infections, ORF7a mRNA expression levels were quantified by qRT-PCR and normalized to infection levels in absence of IgG antibodies (non-treated). Results are shown as mean ±SD from 3 independent experiments. Nonlinear regression is indicated by the purple line. Relative inhibitory concentration of 50% (IC50) values is indicated by the dotted line.

Supplementary Table 1.

Multiple reaction monitoring LC-MS/MS detection of FFAs in S. Signal area of m/z 92 fragments of picolylamine-derivatized PA, OA, LA and AA in S samples were normalized to the mean background signal intensities. Results are shown as mean ± SD from two separate quantifications and at least 2 biological duplicates. n.d. = not detected

	Normalized PA signal	Normalized OA signal	Normalized LA signal	Normalized AA signal
**Native S (D614G)**	1.14 ± 0.21	1.29 ± 0.06	403.24 ± 18.54	n.d.
**ApoS**	1.89 ± 0.21	2.06 ± 0.25	1.19 ± 0.09	n.d.
**ApoS(PA)**	2.20 ± 0.08	2.19 ± 0.06	1.27 ± 0.08	n.d.
**ApoS(OA)**	1.10 ± 0.03	1322.23 ± 15.68	10.96 ± 0.14	n.d.
**ApoS(LA)**	1.47 ± 0.07	1.43 ± 0.04	365.61 ± 0.07	n.d.
**ApoS(AA)**	1.18 ± 0.07	1.13 ± 0.12	1.36 ± 0.05	50.31 ± 1.71
**Background**	1.00 ± 0.28	1.00 ± 0.33	1.00 ± 0.14	n.d.
