# Supplementary material for: Synthetic virions reveal fatty acid-coupled adaptive immunogenicity of SARS-CoV-2 spike glycoprotein
Source: Nat Commun. 2022 Feb 14;13:868. doi: 10.1038/s41467-022-28446-x (PMC8844029; doi:10.1038/s41467-022-28446-x)
Supplement: Supplementary file 3 — Description of Additional Supplementary Information [file 41467_2022_28446_MOESM3_ESM.pdf]

Description of Additional Supplementary Information

Title: Supplementary Movie 1

Description: Cryo-EM tomogram of a MiniV. Scale bar is 180 nm.

Title: Supplementary Movie 2

Description: Epifluorescence microscopy time laps of MCF-7 cells incubated with MiniVs (magenta).
