# Supplementary material for: Synthetic virions reveal fatty acid-coupled adaptive immunogenicity of SARS-CoV-2 spike glycoprotein
Source: Nat Commun. 2022 Feb 14;13:868. doi: 10.1038/s41467-022-28446-x (PMC8844029; doi:10.1038/s41467-022-28446-x)
Supplement: Supplementary file 6 — Reporting summary [file 41467_2022_28446_MOESM6_ESM.pdf]

nature portfolio

Corresponding author(s): Oskar Staufer, Imre Berger, Joachim P. Spatz

Last updated by author(s): Jan 3, 2022

Reporting Summary

Nature Portfolio wishes to improve the reproducibility of the work that we publish. This form provides structure for consistency and transparency in reporting. For further information on Nature Portfolio policies, see our Editorial Policies and the Editorial Policy Checklist.

Statistics

For all statistical analyses, confirm that the following items are present in the figure legend, table legend, main text, or Methods section.

n/a	Confirmed	
☐	☒	The exact sample size (*n*) for each experimental group/condition, given as a discrete number and unit of measurement
☐	☒	A statement on whether measurements were taken from distinct samples or whether the same sample was measured repeatedly
☐	☒	The statistical test(s) used AND whether they are one- or two-sided<br>*Only common tests should be described solely by name; describe more complex techniques in the Methods section.*
☒	☐	A description of all covariates tested
☒	☐	A description of any assumptions or corrections, such as tests of normality and adjustment for multiple comparisons
☐	☒	A full description of the statistical parameters including central tendency (e.g. means) or other basic estimates (e.g. regression coefficient) AND variation (e.g. standard deviation) or associated estimates of uncertainty (e.g. confidence intervals)
☐	☒	For null hypothesis testing, the test statistic (e.g. *F*, *t*, *r*) with confidence intervals, effect sizes, degrees of freedom and *P* value noted<br>*Give P values as exact values whenever suitable.*
☒	☐	For Bayesian analysis, information on the choice of priors and Markov chain Monte Carlo settings
☒	☐	For hierarchical and complex designs, identification of the appropriate level for tests and full reporting of outcomes
☒	☐	Estimates of effect sizes (e.g. Cohen's *d*, Pearson's *r*), indicating how they were calculated

Our web collection on statistics for biologists contains articles on many of the points above.

Software and code

Policy information about availability of computer code

Data collection	Software for data collection is specified in the materials and methods section. Particularly, for data collection based on confocal imaging (Zeiss Zen Blue Edition), plate reader measurements (Tecan Icontrol 2.0.10.0), MassSpec (SCIEX OS, Analyst 1.7 software), cryoTEM (RELION3, IMOD 4.11), protein structure illustration (Mol* Viewer:) and dynamic light scattering (Zetasizer Nano software v3.30) implemented software from the respective hardware manufacturers were used.
Data analysis	For analysis of cryoTEM data, images were aligned using IMOD 4.11 and RELION3 software suite as detailed in the materials and methods section. Microscopy data was analyzed by ImageJ software (2.1.0/1.53).

For manuscripts utilizing custom algorithms or software that are central to the research but not yet described in published literature, software must be made available to editors and reviewers. We strongly encourage code deposition in a community repository (e.g. GitHub). See the Nature Portfolio guidelines for submitting code & software for further information.

Data

Policy information about availability of data

All manuscripts must include a data availability statement. This statement should provide the following information, where applicable:

- Accession codes, unique identifiers, or web links for publicly available datasets
- A description of any restrictions on data availability
- For clinical datasets or third party data, please ensure that the statement adheres to our policy

The data generated in this study are available in the main text, the supplementary materials, the source data file or the corresponding authors upon reasonable request. Source data are provided as separate source data file. Protein structures used in this study were retrieved from the protein data bank under the accession codes 7BNN, 6ZB5 and 7A97.

Field-specific reporting

Please select the one below that is the best fit for your research. If you are not sure, read the appropriate sections before making your selection.

[x] Life sciences [ ] Behavioural & social sciences [ ] Ecological, evolutionary & environmental sciences

For a reference copy of the document with all sections, see nature.com/documents/nr-reporting-summary-flat.pdf

Life sciences study design

All studies must disclose on these points even when the disclosure is negative.

Sample size	No sample size calculations were performed beforehand but samples sizes were chosen according to preliminary measurements and testing in this study. An appropriate number of technical and biological replicates were generated under similar conditions yielding statistically significant results between groups. Moreover, sample size was determined to be adequate based on previously published researches. For plate reader assessment of retention, three biological replicates were performed to test for variation in samples handling, cell plating, measurements variation and cell layer homogeneity.
Data exclusions	No data were excluded from the analysis
Replication	All retention measurements were verified in biological triplicates. Dynamic light scattering measurements were performed with at least 10 runs per sample and in three consecutive measurements. For dynamic light scattering experiments, all attempts of replication were successful. Assessment of competition for infection between SARS-CoV-2 viruses and MiniVs were performed in three independent experiments. MassSpec assessment was performed with at least two biological triplicates and at least two consecutive injections. No non-replicable experiments were excluded or reported in this study.
Randomization	No allocation in experimental groups was performed.
Blinding	Blinding was not relevant for the presented study, as data collection was performed automatically, and the data under investigation need to be known.

Reporting for specific materials, systems and methods

We require information from authors about some types of materials, experimental systems and methods used in many studies. Here, indicate whether each material, system or method listed is relevant to your study. If you are not sure if a list item applies to your research, read the appropriate section before selecting a response.

Materials & experimental systems

n/a	Involved in the study	
[ ]	[x]	Antibodies
[ ]	[x]	Eukaryotic cell lines
[x]	[ ]	Palaeontology and archaeology
[x]	[ ]	Animals and other organisms
[x]	[ ]	Human research participants
[x]	[ ]	Clinical data
[x]	[ ]	Dual use research of concern

Methods

n/a	Involved in the study	
[x]	[ ]	ChIP-seq
[x]	[ ]	Flow cytometry
[x]	[ ]	MRI-based neuroimaging

Antibodies

Antibodies used	Human IgG COVID-19 convalescent plasma fractionated purified and lyophilized was purchased from Innovative Research, USA. Catalog number IHUCOVIGGGFLYSOUG (expire date 04/082026).<br>Anti SARS23 CoV-2 S, clone CR3022 (FITC) was purchased from Novus Biologiclas, Germany. Catalog number NBP2-90980F, LotT2026COI-020821<br>Dilutions or concentration of each antibody or antibody mixture are indicated in the figures or figure legends.
Validation	All antibodies were tested and verified by the manufacturers. CR3022 was generated by sequencing peripheral blood lymphocytes of a patient exposed to SARS-CoV. Validation results were published by Atyeo, C. et al. Dissecting strategies to tune the therapeutic potential of SARS-CoV-2- specific monoclonal antibody CR3022. 5 JCI Insight 6, doi:10.1172/jci.insight.143129 (2021). Human IgG COVID-19 convalescent plasma fractionated purified and lyophilized was obtained from patients with PCR positive SRAS-CoV-2 status and serum was collected two weeks after symptom onset.

Eukaryotic cell lines

Policy information about cell lines

Cell line source(s)	MCF-7 and A549 cells were obtained from the American Type Culture Collection.
Authentication	None of the cell lines were authenticated by the authors but the commercial providers of the cell lines verified the authenticity.
Mycoplasma contamination	All cell lines were tested mycoplasma negative.
Commonly misidentified lines (See ICLAC register)	MCF-7 cells, listed as commonly misidentified lines, were used in this study. Cells were directly obtained from the American Tissue Culture Collection (ATCC), providing STR profiling, and non of the common contaminating cell lines were culture in the lab.
